# Identification of Potential ceRNA Network and Patterns of Immune Cell Infiltration in Systemic Sclerosis-Associated Interstitial Lung Disease

**DOI:** 10.3389/fcell.2021.622021

**Published:** 2021-06-17

**Authors:** Qiuhong Wu, Yang Liu, Yan Xie, Shixiong Wei, Yi Liu

**Affiliations:** ^1^Department of Rheumatology and Immunology, West China Hospital, Sichuan University, Chengdu, China; ^2^Lung Cancer Center, West China Hospital, Sichuan University, Chengdu, China; ^3^Rare Diseases Center, West China Hospital, Sichuan University, Chengdu, China; ^4^Institute of Immunology and Inflammation, Frontiers Science Center for Disease-Related Molecular Network, West China Hospital, Sichuan University, Chengdu, China

**Keywords:** systemic sclerosis, interstitial lung disease, ceRNA, immune cell infiltration, GEO

## Abstract

**Purpose:**

Systemic sclerosis-associated interstitial lung disease (SSc-ILD) is one of the most severe complications of systemic sclerosis (SSc) and is the leading cause of SSc-related deaths. However, the precise pathogenesis of pulmonary fibrosis in SSc-ILD remains unknown. This study aimed to evaluate the competing endogenous RNA (ceRNA) regulatory network and immune cell infiltration patterns in SSc-ILD.

**Methods:**

One microRNA (miRNA) and three messenger RNA (mRNA) microarray datasets were obtained from the Gene Expression Omnibus (GEO) database. Then, the differentially expressed miRNAs (DEmiRs) and mRNAs (DEMs) between SSc-ILD patients and normal controls were identified, respectively, followed by the prediction of the target genes and target lncRNAs of DEmiRs. The overlapping genes between DEmiRs target genes and DEMs were identified as core mRNAs to construct the ceRNA network. In addition, the “Cell Type Identification by Estimating Relative Subsets of Known RNA Transcripts (CIBERSORT)” algorithm was used to analyze the composition of infiltrating immune cells in lung tissues of SSc-ILD patients and controls, and differentially expressed immune cells were recognized. The correlation between immune cells and core mRNAs was evaluated by Pearson correlation analysis.

**Results:**

Totally, 42 SSc-ILD lung tissues and 18 normal lung tissues were included in this study. We identified 35 DEmiRs and 142 DEMs and predicted 1,265 target genes of DEmiRs. Then, 9 core mRNAs related to SSc-ILD were recognized, which were the overlapping genes between DEmiRs target genes and DEMs. Meanwhile, 9 DEmiRs related to core mRNAs were identified reversely, and their target lncRNAs were predicted. In total, 9 DEmiRs, 9 core mRNAs, and 51 predicted lncRNAs were integrated to construct the ceRNA regulatory network of SSc-ILD. In addition, 9 types of immune cells were differentially expressed in lung tissues between SSc-ILD patients and controls. Some core mRNAs, such as *COL1A1*, *FOS*, and *EDN1*, were positively or negatively correlated with the number of infiltrating immune cells.

**Conclusion:**

This is the first comprehensive study to construct the potential ceRNA regulatory network and analyze the composition of infiltrating immune cells in lung tissues of SSc-ILD patients, which improves our understanding of the pathogenesis of SSc-ILD.

## Introduction

Systemic sclerosis (SSc) is a systemic autoimmune disease characterized by immune dysregulation, vasculopathy, and localized or diffused skin fibrosis. As a rare disease, the incidence of SSc is very low (10–50 per million people per year), but the mortality rate of SSc is relatively higher than other rheumatic diseases (Allanore et al., 2015; Denton and Khanna, 2017). Systemic sclerosis-associated interstitial lung disease (SSc-ILD) is a common SSc complication, which is the main cause of SSc-related deaths (Perelas et al., 2020). However, the treatment options of SSc-ILD are limited, partly because of the fact that the precise mechanisms of the development of pulmonary fibrosis in SSc-ILD have not been clearly elucidated. Thus, exploring potential pathogenesis in SSc-ILD and finding effective biomarkers for the diagnosis and treatment of SSc-ILD are quite necessary.

Non-coding RNAs (ncRNAs) account for nearly 98% of the human genome. Although ncRNAs do not code for proteins, they play significant roles in maintaining normal development and physiological functions by regulating gene expression at both the transcriptional and post-transcriptional levels (Esteller, 2011; Cech and Steitz, 2014). MicroRNAs (miRNAs), as a subgroup of short ncRNAs, are evolutionarily conserved single-stranded RNA molecules, which approximately contain 19–25 nucleotides. They can negatively regulate post-transcriptional gene expression by pairing with complementary sequences in the 3′-untranslated region (3′-UTR) of the target messenger RNAs (mRNAs), resulting in target gene degradation or translation inhibition. miRNAs are involved in a variety of cellular biological processes, such as cell proliferation, apoptosis, and differentiation (Bartel, 2004, 2009; Valencia-Sanchez et al., 2006). And accumulating evidences have demonstrated that the dysregulation of miRNAs was associated with the development of many human diseases, such as cancers, neurological disorders, cardiovascular disorders, as well as rheumatic diseases (Nakasa et al., 2008; Esteller, 2011; Christmann et al., 2016; Young et al., 2017). For instance, Nakasa et al. (2008) demonstrated that miR-146a/b was highly expressed in synovial tissues of rheumatoid arthritis (RA). Christmann et al. (2016) found that the expression of miR-155 was related to progressive SSc-ILD. Therefore, miRNAs can be identified as potential biomarkers for the diagnosis, treatment and prognosis of diseases.

Long non-coding RNAs (lncRNAs) are defined as a type of ncRNAs that are longer than 200 nucleotides in length, and they perform multilevel regulatory functions in gene expression, such as transcription, translation and epigenetics. In addition, lncRNAs play pivotal roles in maintaining cellular homeostasis, and differential expression patterns of lncRNAs have been found in many human diseases (Mercer et al., 2009; Li et al., 2013; Shi et al., 2013). Salmena et al. (2011) put forward a competing endogenous RNA (ceRNA) hypothesis, in which lncRNAs, mRNAs, and other RNAs were able to compete with each other as ceRNAs to bind to miRNAs through sharing one or more miRNA response elements (MREs). lncRNAs can serve as endogenous molecular sponges for miRNAs to indirectly regulate the expression of mRNAs, and the ceRNA network links the function of ncRNAs to the function of protein-coding mRNAs (Salmena et al., 2011; Tay et al., 2014). In recent years, many studies have indicated that the dysregulation of lncRNA-miRNA-mRNA networks was closely related to the pathogenesis of many human diseases, such as cancers (Wang et al., 2016; Zhang et al., 2016). Nevertheless, the potential ceRNA regulatory network in SSc-ILD has not been explored.

To be mentioned, the occurrence and progression of SSc-ILD is a complicated process involving multiple biological mechanisms, such as autoimmunity, fibrosis, inflammation, and vascular injury (Ciechomska et al., 2015; Furue et al., 2017; Brown and O’Reilly, 2019; Perelas et al., 2020). Immune cell infiltration is closely associated with the fibrosis of SSc patients. Many immune cells, such as T cells, B cells, monocytes, dendritic cells (DCs), macrophages, and mast cells, have been indicated in the pathogenesis of fibrosis in SSc (Brown and O’Reilly, 2019). For instance, Kalogerou et al. (2005) found that the expression levels of CD69, the early T cell activation marker, were increased in skin biopsy samples of SSc patients, implying that T cells might be included in the process of skin fibrosis. Francois et al. (2013) indicated that B cells played important roles in the production of collagens by dermal fibroblasts and could be the inducers of fibrosis in SSc. However, in SSc-ILD, the landscape of immune cell infiltration has not been fully elucidated.

In this study, we download one miRNA microarray dataset (GSE81293) and three mRNA microarray datasets (GSE81292, GSE48149, and GSE76808) from the Gene Expression Omnibus (GEO) database and identified differentially expressed miRNAs (DEmiRs) and differentially expressed mRNAs (DEMs) in lung tissues between SSc-ILD patients and normal controls. Then, the target genes and target lncRNAs of DEmiRs were predicted. The overlapping genes between DEmiRs target genes and DEMs were identified as core mRNAs to construct the potential ceRNA regulatory network of SSc-ILD. Meanwhile, the algorithm “Cell Type Identification by Estimating Relative Subsets of Known RNA Transcripts (CIBERSORT)” was used to analyze the different patterns of immune cell infiltration between SSc-ILD lung tissues and normal lung tissues. Moreover, the co-expression patterns of immune cells and core mRNAs were also evaluated. This study aimed to explore the potential ceRNA-related pathogenesis of SSc-ILD and identify the underlying immune gene signature of SSc-ILD.

## Materials and Methods

### Microarray Data Download and Processing

A total of one miRNA microarray dataset GSE81293 (Christmann et al., 2016) and three mRNA microarray datasets GSE81292 (Christmann et al., 2016), GSE48149 (Hsu et al., 2011; Renaud et al., 2020), and GSE76808 (Christmann et al., 2014) from lung biopsy tissues of SSc-ILD patients and controls were downloaded from the National Center for Biotechnology Information (NCBI) GEO database^1^. The miRNA microarray dataset GSE81293 was based on the GPL16384 platform ([miRNA-3] Affymetrix Multispecies miRNA-3 Array) and included 15 SSc-ILD samples and 5 normal control samples. And a total of 42 SSc-ILD lung specimens and 18 normal lung tissues were enrolled from three mRNA microarray datasets GSE81292, GSE48149, and GSE76808, whose detection platforms were GPL18991 ([HG-U133A_2] Affymetrix Human Genome U133A 2.0 Array), GPL16221 (Illumina HumanRef-8 v3.0 expression beadchip), and GPL571 ([HG-U133A_2] Affymetrix Human Genome U133A 2.0 Array), respectively (Table 1).

**TABLE 1:** Details of miRNA and mRNA datasets of patients with SSc-ILD.

**Type**	**GEO accession**	**Platform**	**Sample organism**	**Experiment type**	**Samples (lung tissues), *n***	
					**SSc-ILD patients**	**Controls**
miRNA	GSE81293	GPL16384	Homo sapiens	Non-coding RNA profiling by array	15	5
mRNA	GSE81292	GPL18991	Homo sapiens	Expression profiling by array	15	5
	GSE48149	GPL16221	Homo sapiens	Expression profiling by array	13	9
	GSE76808	GPL571	Homo sapiens	Expression profiling by array	14	4

**miRNA, microRNA; mRNA, messenger RNA; SSc-ILD, systemic sclerosis-associated interstitial lung disease.**

Each probe expression matrix was matched with its platform annotation, and the probe IDs were transformed into homologous gene symbols. All the expression data were normalized and log2 transformed. If one gene had more than one probe expression data, the average value was taken for further analysis. Then, the expression matrixes of three mRNA microarray datasets GSE81292, GSE48149, and GSE76808 were merged into one, and the heterogeneity caused by different experimental platforms and batches was eliminated, which was conducted using “sva” package of R software (v.4.0.5) (Leek et al., 2020).

### Differential Expression Analysis

The miRNAs and mRNAs that were differentially expressed between SSc-ILD lung tissues and normal lung tissues were identified by “limma” package of R software (Ritchie et al., 2015). *P*-values were adjusted by the Benjamini-Hochberg (BH) false discovery rate (FDR) method (Glickman et al., 2014). RNAs that meet the screening criteria [| log2 fold change (FC)| > 1, and FDR adjusted *P*-value (*q*-value) < 0.05] were considered as DEmiRs or DEMs. Then, heat maps and volcano plots were drawn using “pheatmap” and “ggplot2” packages of R software to depict these differentially expressed RNAs.

### Gene Ontology (GO) and Pathway Enrichment Analysis

In order to explore the potential biological mechanisms of DEMs in SSc-ILD, GO enrichment analysis [including biological process (BP), cellular component (CC), and molecular function (MF)] and Kyoto Encyclopedia of Genes and Genomes (KEGG) pathway enrichment analysis were conducted using R “ClusterProfiler” and “org.Hs.eg.db” packages (Yu et al., 2012). As for DEmiRs, the GO and biological pathway enrichment analysis were carried out using the FunRich software (v.3.1.3) (Pathan et al., 2015). The *q*-value < 0.05 was considered statistically significant.

### Construction of the Protein-Protein Interaction (PPI) Network and Hub Gene Analysis

The Search Tool for the Retrieval of Interacting Genes (STRING) online database^2^ was used to analyze the PPI network for the DEMs with interaction score > 0.7 (Szklarczyk et al., 2019). Then, the PPI network was constructed and visualized using Cytoscape software (v.3.8.1), and the top 10 hub genes were identified by the Cytoscape’s cytoHubba plugin according to the number of connections (Chin et al., 2014).

### Prediction of DEmiRs Target Genes and Identification of Core mRNAs

The DEmiRs target genes were predicted using three databases including TargetScan (Agarwal et al., 2015), miRTarBase (Huang et al., 2020), and miRDB (Chen and Wang, 2020). Only those genes that concurrently existed in all three databases were considered as the candidate targets of DEmiRs. Subsequently, the intersection of DEmiRs target genes and DEMs was analyzed using the Venny 2.1.0 online database^3^, and the overlapping genes were identified as core mRNAs related to SSc-ILD.

### Prediction of DEmiRs Target lncRNAs and Construction of the ceRNA Network

The DIANA-LncBase v.2 prediction module^4^ was used to predict the target lncRNAs of DEmiRs related to core mRNAs (Paraskevopoulou et al., 2016). The lncRNAs that meet the filter criteria (prediction interaction score > 0.99 and lncRNA tissue type: lung) were selected as potential DEmiRs target lncRNAs. Then, DEmiRs regulated for both lncRNAs and core mRNAs were selected for the construction of ceRNA regulatory network, which was visualized by Cytoscape software (v.3.8.1).

### Analysis of Immune Cell Infiltration

To analyze the proportion of 22 infiltrating immune cells in lung tissues of SSc-ILD patients and normal controls, the mRNA expression data were uploaded to the online platform of CIBERSORT^5^, with 1,000 permutations applied to the algorithm (Newman et al., 2015). The CIBERSORT results with output *P*-values < 0.05 were considered eligible and accurate for further analysis. Subsequently, the Wilcoxon rank-sum test was performed to assess the differential composition of infiltrating immune cells between SSc-ILD patients and controls, which was visualized by “pheatmap” and “vioplot” packages in R software. Two-sided *P* < 0.05 was considered statistically significant. Furthermore, the correlation among 22 immune cell subtypes and the relationship between differentially expressed immune cells and core mRNAs were evaluated using Pearson correlation analysis.

## Results

### Identification of DEmiRs and DEMs in SSc-ILD

**FIGURE 1:**
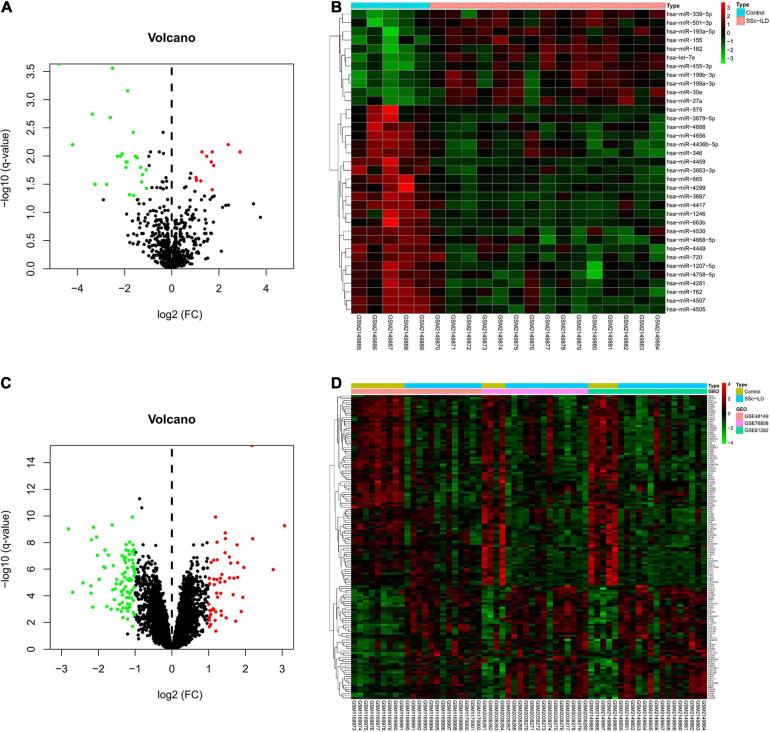
DEmiRs and DEMs in lung tissues between SSc-ILD patients and normal controls. **(A)** The volcano plot of DEmiRs. **(B)** The heat map of DEmiRs. **(C)** The volcano plot of DEMs. **(D)** The heat map of DEMs. DEmiRs, differentially expressed microRNAs; DEMs, differentially expressed messenger RNAs; SSc-ILD, systemic sclerosis-associated interstitial lung disease.

In total, 42 SSc-ILD lung samples and 18 normal lung tissues from GEO database were included in this study, and the details of these datasets (one miRNA microarray dataset and three mRNA microarray datasets) were shown in Table 1. In the miRNA microarray dataset GSE81293, 35 DEmiRs were identified in lung tissues of SSc-ILD patients compared with matched normal lung tissues, of which 11 DEmiRs were upregulated and 24 DEmiRs were downregulated in SSc-ILD. The volcano plot and heat map of these DEmiRs were shown in Figures 1A,B. As for the merged mRNA microarray dataset, a total of 142 DEMs were obtained between SSc-ILD patients and normal controls, including 53 upregulated DEMs and 89 downregulated DEMs in SSc-ILD, which were shown by volcano plot and heat map in Figures 1C,D.

### GO and Pathway Enrichment Analysis of DEmiRs and DEMs in SSc-ILD

**FIGURE 2:**
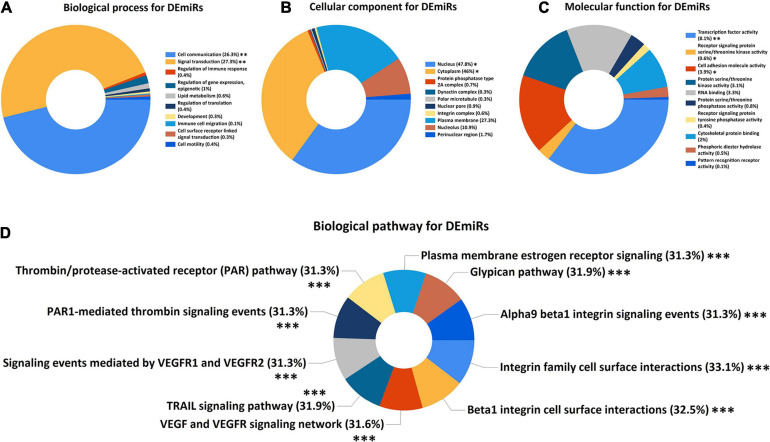
GO and biological pathway enrichment analysis of DEmiRs. **(A)** Biological process for DEmiRs. **(B)** Cellular component for DEmiRs. **(C)** Molecular function for DEmiRs. **(D)** Biological pathway for DEmiRs. GO, Gene Ontology; DEmiRs, differentially expressed microRNAs. The asterisks represented the statistical *q*-values (^∗^*q*-value < 0.05; ^∗∗^*q*-value < 0.01; ^∗∗∗^*q*-value < 0.001).

In order to explore the potential mechanisms of DEmiRs and DEMs in SSc-ILD, GO and pathway enrichment analysis were performed. Figure 2 showed the top 10 enriched GO terms including BP, CC, and MF and the top 10 enriched biological pathways of DEmiRs. The BP terms of these DEmiRs were significantly enriched in cell communication (*q*-value < 0.01) and signal transduction (*q*-value < 0.01). Nucleus (*q*-value < 0.05) and cytoplasm (*q*-value < 0.05) were two significantly enriched CC terms. And in the MF group, DEmiRs were primarily enriched in transcription factor activity (*q*-value < 0.01), cell adhesion molecule activity (*q*-value < 0.05), and receptor signaling protein serine/threonine kinase activity (*q*-value < 0.05) (Figures 2A–C). As for the biological pathway enrichment analysis of DEmiRs, they were significantly enriched in some fibrosis-related signaling pathways, such as integrin family cell surface interactions, beta1 integrin cell surface interactions, TRAIL signaling pathway, and VEGF and VEGFR signaling network (all *q*-values < 0.001) (Figure 2D).

**FIGURE 3:**
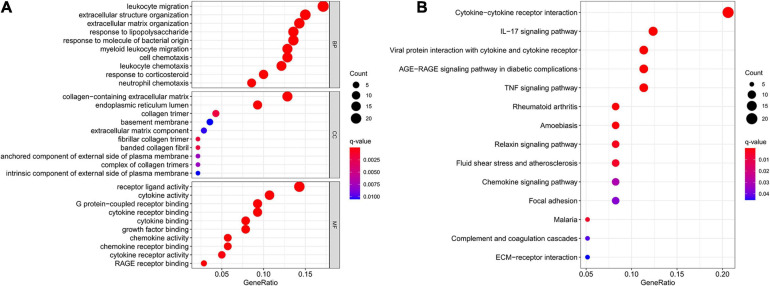
GO and KEGG enrichment analysis of DEMs. **(A)** GO enrichment analysis of DEMs. **(B)** KEGG enrichment analysis of DEMs. GO, Gene Ontology; KEGG, KyotoEncyclopedia of Genes and Genomes; DEMs, differentially expressed messenger RNAs.

The results of GO and KEGG enrichment analysis of DEMs were shown in Figure 3. In the BP category, DEMs were mainly enriched in leukocyte migration, extracellular structure organization, extracellular matrix organization, response to lipopolysaccharide, and response to molecule of bacterial origin. Collagen-containing extracellular matrix, endoplasmic reticulum lumen, and collagen trimer were the top three enriched terms of CC. Regarding MF, DEMs were significantly enriched in receptor ligand activity, cytokine activity, G protein-coupled receptor binding, cytokine receptor binding, and cytokine binding. In addition, the KEGG analysis indicated that DEMs were significantly enriched in 14 pathways, such as cytokine-cytokine receptor interaction, IL-17 signaling pathway, viral protein interaction with cytokine and cytokine receptor, AGE-RAGE signaling pathway in diabetic complications, and TNF signaling pathway.

### PPI Network Construction and Hub Genes Identification

**FIGURE 4:**
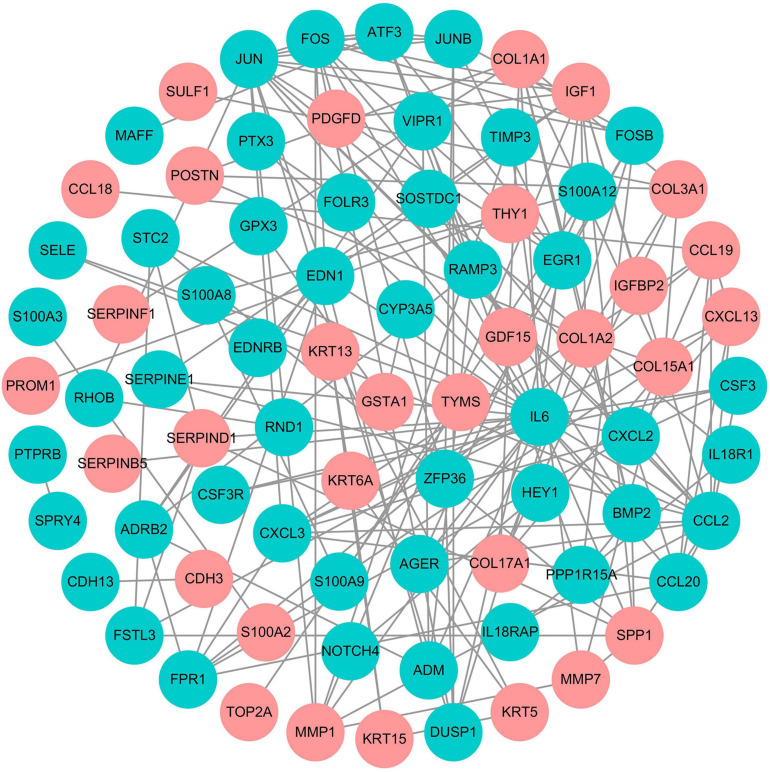
The PPI network of DEMs in SSc-ILD. The red dots represent the upregulated genes, and the green dots represent the downregulated genes. PPI, protein-protein interaction; DEMs, differentially expressed messenger RNAs; SSc-ILD, systemic sclerosis-associated interstitial lung disease.

The PPI network containing 80 nodes and 164 edges was constructed based on the STRING online database and visualized by Cytoscape software. Subsequently, the top 10 hub genes, including *IL6* (interleukin 6), *JUN* (Jun proto-oncogene), *CCL2* (C-C motif chemokine ligand 2), *FOS* (Fos proto-oncogene), *IGF1* (insulin like growth factor 1), *CXCL2* (C-X-C motif chemokine ligand 2), *ATF3* (activating transcription factor 3), *EDN1* (endothelin 1), *COL1A1* (collagen type I alpha 1 chain), and *SPP1* (secreted phosphoprotein 1), were identified using the Cytoscape’s cytoHubba plugin according to the number of connections (Figure 4).

### Identification of Core mRNAs and Construction of the ceRNA Network in SSc-ILD

**FIGURE 5:**
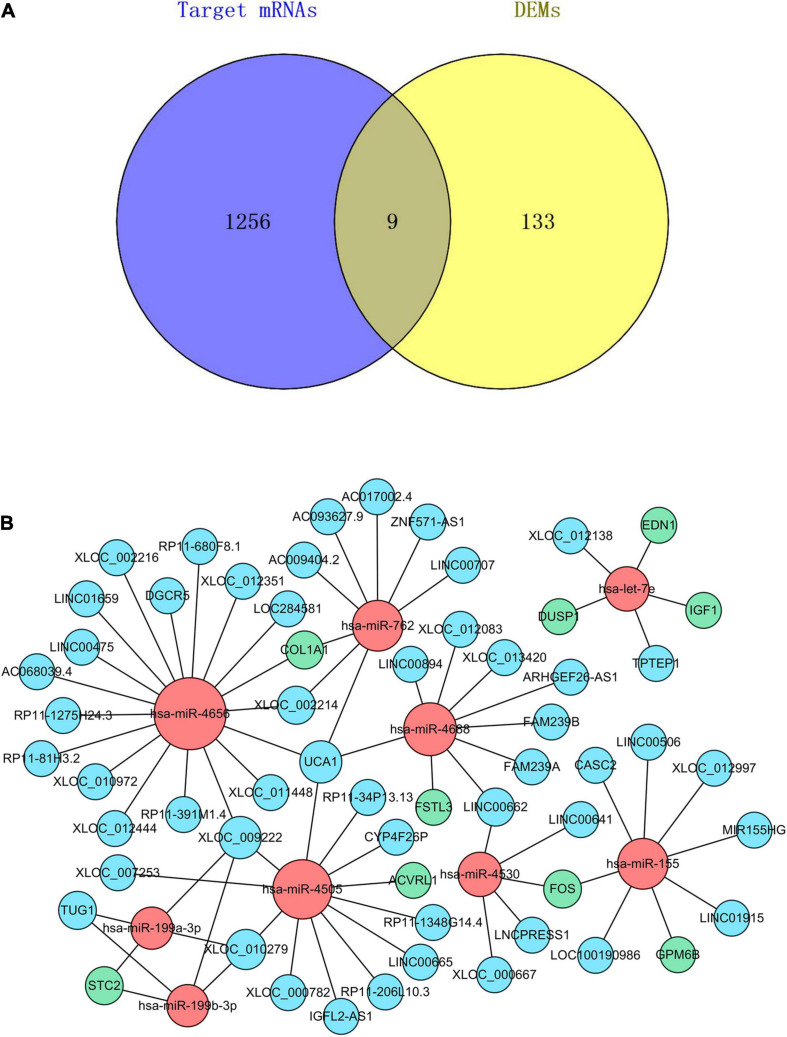
The core mRNAs identification and ceRNA regulatory network in SSc-ILD. **(A)** The intersection between DEMs and the target genes of DEmiRs. **(B)** The ceRNA regulatory network. The red dots represent DEmiRs, the green dots represent core mRNAs, and the blue dots represent predicted lncRNAs. mRNAs, messenger RNAs; ceRNA, competing endogenous RNA; SSc-ILD, systemic sclerosis-associated interstitial lung disease; DEMs, differentially expressed mRNAs; DEmiRs, differentially expressed microRNAs; lncRNAs, long non-coding RNAs.

We used three databases, TargetScan, miRTarBase, and miRDB, to predict the target genes of DEmiRs. There were a total of 1,265 target genes that coexisted in all the three databases (Supplementary Table 1). Then, the Venn method was used to analyze the intersection between DEMs and these DEmiRs target genes. As a result, 9 overlapping core mRNAs associated with SSc-ILD were identified (Figure 5A). According to these 9 core mRNAs, 9 DEmiRs were recognized reversely. Subsequently, we used the DIANA-LncBase v.2 prediction module to explore the target lncRNAs that could potentially bind to these 9 DEmiRs related to core mRNAs. These 9 DEmiRs could interact not only with lncRNAs but also with core mRNAs, which were selected for the construction of ceRNA regulatory network. In total, 9 DEmiRs, 9 core mRNAs, and 51 predicted lncRNAs were integrated to construct the potential ceRNA network of SSc-ILD, which included 69 nodes and 73 edges (Table 2 and Figure 5B).

**TABLE 2:** The DEmiRs, core mRNAs, and predicted lncRNAs in the ceRNA network.

**miRNA**	**Core mRNA**	**lncRNA**
hsa-let-7e	DUSP1, EDN1, IGF1	XLOC_012138, TPTEP1
hsa-miR-155	FOS, GPM6B	LOC100190986, LINC00506, MIR155HG, XLOC_012997, LINC01915, CASC2
hsa-miR-199a-3p	STC2	XLOC_009222, TUG1, XLOC_010279
hsa-miR-199b-3p	STC2	XLOC_009222, TUG1, XLOC_010279
hsa-miR-4505	ACVRL1	CYP4F26P, UCA1, RP11-34P13.13, XLOC_009222, RP11-206L10.3, XLOC_010279, XLOC_007253, XLOC_000782, LINC00665, IGFL2-AS1, RP11-1348G14.4
hsa-miR-4530	FOS	XLOC_000667, LNCPRESS1, LINC00662, LINC00641
hsa-miR-4656	COL1A1	UCA1, RP11-81H3.2, DGCR5, XLOC_009222, RP11-391M1.4, LINC01659, AC068039.4, XLOC_002214, LOC284581, RP11-1275H24.3, XLOC_002216, XLOC_010972, LINC00475, RP11-680F8.1, XLOC_012444, XLOC_012351, XLOC_011448
hsa-miR-4688	FSTL3	UCA1, XLOC_012083, LINC00894, ARHGEF26-AS1, FAM239A, FAM239B, XLOC_013420, LINC00662
hsa-miR-762	COL1A1	UCA1, AC093627.9, XLOC_002214, ZNF571-AS1, AC009404.2, LINC00707, AC017002.4

**DEmiRs, differentially expressed microRNAs; mRNAs, messenger RNAs; ceRNA, competing endogenous RNA.**

### Composition of Infiltrating Immune Cells in SSc-ILD

**FIGURE 6:**
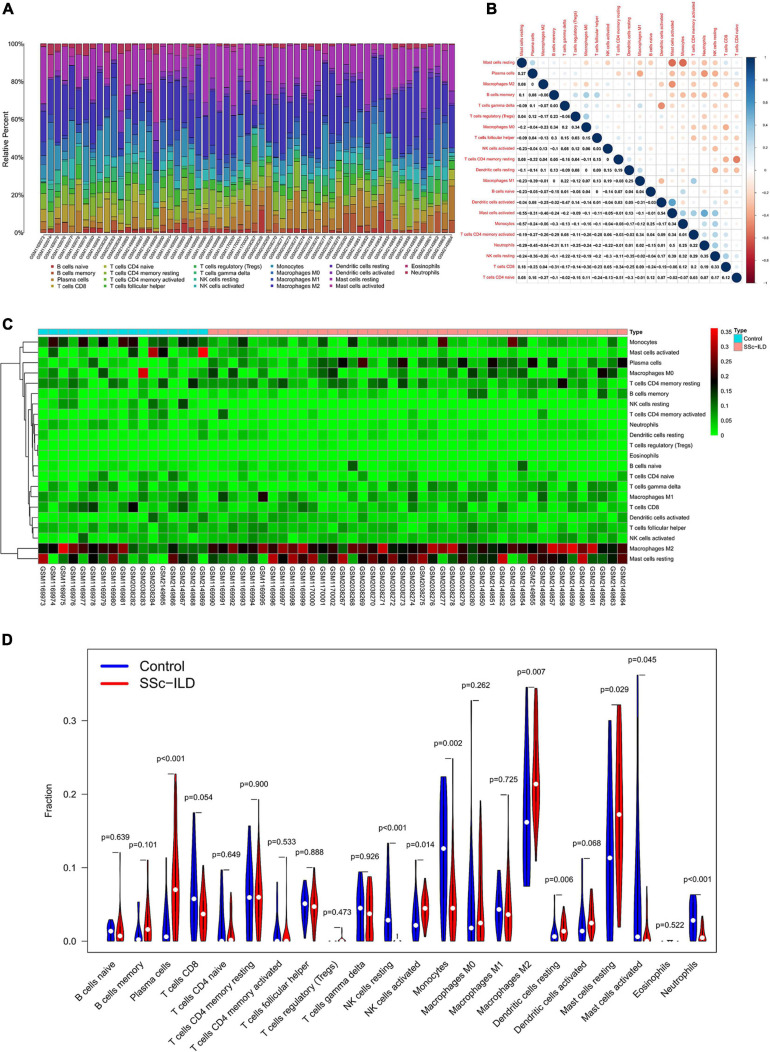
Composition of infiltrating immune cells in lung tissues. **(A)** The distribution of immune cell types in each sample. **(B)** The correlation among immune cell types. **(C)** The heat map of infiltrating immune cells. **(D)** The violin plot of infiltrating immune cells.

The composition of 22 infiltrating immune cells in lung tissues of SSc-ILD patients and normal controls were estimated by the CIBERSORT algorithm (Figure 6A). Since the output *P*-value of GSM2038281 was greater than 0.05 (*P* = 0.077), it was excluded for further analysis. The distribution of 22 immune cell types in each sample varied significantly, of which M2 macrophages accounted for the most enriched proportion. The relationships among 22 immune cells were indicated in Figure 6B. Activated mast cells were negatively correlated with resting mast cells (*R* = −0.55), whereas positively correlated with activated DCs (*R* = 0.54). Naive CD4^+^ T cells had a negative correlation with resting memory CD4^+^ T cells (*R* = −0.51), and monocytes were negatively correlated with resting mast cells (*R* = −0.57). Other immune cell subpopulations were weakly to moderately correlated.

The differential proportion of infiltrating immune cells between SSc-ILD patients and controls was analyzed by Wilcoxon rank-sum test, and the results showed that 9 types of immune cells, plasma cells, resting NK cells, activated NK cells, monocytes, M2 macrophages, resting DCs, resting mast cells, activated mast cells, and neutrophils, were differentially expressed. Specifically, plasma cells, activated NK cells, M2 macrophages, resting DCs, and resting mast cells were upregulated in lung tissues of SSc-ILD patients (Figures 6C,D).

### Co-expression Patterns of Immune Cells and Core mRNAs

**FIGURE 7:**
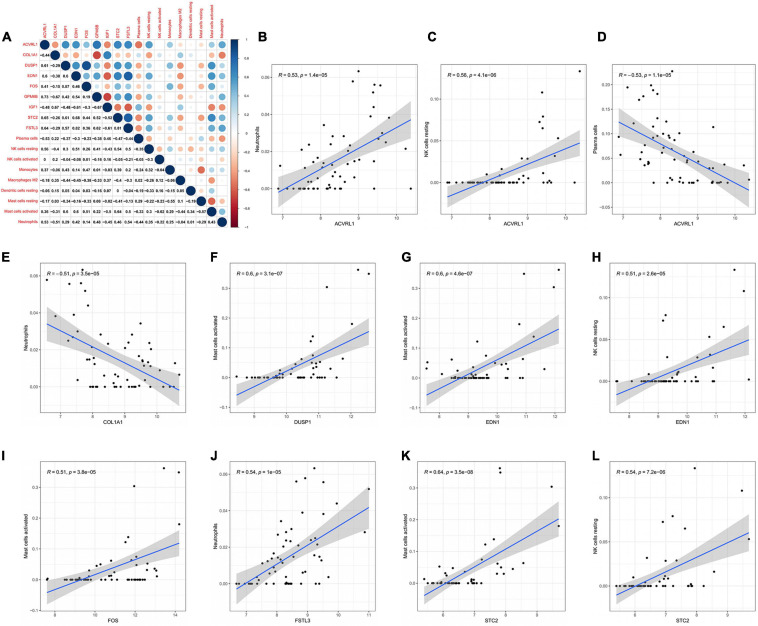
Co-expression patterns of differential immune cells and core mRNAs. **(A)** The correlation between core mRNAs and differential immune cells. **(B–L)** The significantly correlated pairs with correlation coefficient > 0.5 and *P* < 0.001. mRNAs, messenger RNAs.

The correlation between core mRNAs and differentially expressed immune cells was estimated via Pearson correlation analysis, and the significantly correlated pairs with correlation coefficient > 0.5 and *P* < 0.001 were shown in Figure 7. The *ACVRL1* (activin A receptor like type 1) gene was positively correlated with neutrophils and resting NK cells (*R* = 0.53 and *R* = 0.56, respectively), but negatively correlated with plasma cells (*R* = −0.53). The *DUSP1* (dual specificity phosphatase 1) gene and *FOS* gene were both positively correlated with activated mast cells (*R* = 0.6 and *R* = 0.51, respectively). And neutrophils could be negatively regulated by the *COL1A1* gene (*R* = −0.51) but be positively regulated by the *FSTL3* (follistatin like 3) gene (*R* = 0.54). In addition, both the *EDN1* gene and the *STC2* (stanniocalcin 2) gene had a positive correlation with activated mast cells (*R* = 0.6 and *R* = 0.64, respectively) and resting NK cells (*R* = 0.51 and *R* = 0.54, respectively).

## Discussion

SSc-ILD is one of the most severe complications of SSc and is the leading cause of SSc-related deaths. Although significant progress has been achieved in the study of the pathogenesis of SSc-ILD, the detailed molecular mechanisms of the lung fibrosis in SSc-ILD remain largely unknown. Therefore, in order to improve the diagnosis and treatment of SSc-ILD, it is of vital significance to explore the potential molecular mechanisms and identify effective biomarkers with high specificity and sensitivity. In recent years, the hypothesis of ceRNA network has greatly aroused the interest of researchers, and it makes the link between protein-coding mRNAs and non-coding RNAs. Based on the ceRNA hypothesis, lncRNAs can act as miRNA sponges to influence the expression of mRNAs (Salmena et al., 2011; Tay et al., 2014). In this study, in order to explore the potential pathogenesis of SSc-ILD, we constructed the ceRNA regulatory network of SSc-ILD based on 4 microarray datasets.

miRNAs, as post-transcription regulators, play important roles in affecting the expression of downstream target genes, which in turns to participate in a variety of physiological and pathological processes (Valencia-Sanchez et al., 2006; Bartel, 2009). In recent years, the dysregulation of miRNA expression has been found to be related to the occurrence and development of many diseases including SSc-ILD. The expression of miRNAs can be used as an efficient biomarker for the diagnosis and prognosis of diseases. In fact, several miRNAs have been found to be associated with the pathogenesis of SSc fibrosis (Henry et al., 2019). Christmann et al. demonstrated that the expression levels of miR-155 in lung tissues were associated with progressive SSc-ILD. In the bleomycin-induced pulmonary fibrosis model, miR-155 knockout mice developed milder lung fibrosis when compared with wild-type mice (Christmann et al., 2016). Makino et al. indicated that let-7a expression levels were significantly decreased in both the skin and the serum of SSc patients, and the overexpression or inhibition of the expression levels of let-7a in human or mouse skin fibroblasts could regulate the type I collagen expression. Moreover, the overexpression of let-7a in the skin could attenuate the skin fibrosis induced by bleomycin in mice (Makino et al., 2013). Maurer et al. (2010) found that miR-29a had a strong relationship with SSc fibrosis, in which miR-29a was significantly downregulated in SSc skins and fibroblasts, and it could also regulate the expression levels of type I and type III collagens. In the current study, we identified some DEmiRs related to SSc-ILD. Additionally, these DEmiRs were significantly enriched in some well-known signaling pathways that have been proved to be associated with fibrosis, such as integrin family cell surface interactions, beta1 integrin cell surface interactions, TRAIL signaling pathway, and VEGF and VEGFR signaling network (all *q*-values < 0.001), indicating our results were credible.

Furthermore, we integrated three mRNA microarray data to identify the DEMs and uncovered the underlying PPI network and hub genes in SSc-ILD. Meanwhile, the DEmiRs target genes were predicted using three distinct prediction databases. Then, 9 overlapping core mRNAs associated with SSc-ILD were identified, which were not only the target genes of DEmiRs, but also the DEMs between SSc-ILD and controls. Among them, four core mRNAs, *COL1A1*, *IGF1*, *EDN1*, and *FOS*, were also the hub genes existed in the PPI network.

*COL1A1* gene is a fibrosis-related gene that encodes the alpha1 chains of type I collagen, which is the most common extracellular matrix (ECM) protein in tissues. Although the precise mechanisms of fibrosis have not been elucidated, type I collagens play significant roles in the development of tissue fibrosis in SSc (Hitraya and Jimenez, 1996; Jimenez and Saitta, 1999). In fact, a lot of studies have found that the expression levels of collagen corresponding genes including *COL1A1* were increased in SSc fibroblasts, and inhibiting collagen genes expression or collagen proteins production could be an effective treatment for the tissue fibrosis of SSc (Hitraya and Jimenez, 1996; Jimenez and Saitta, 1999; Sandorfi et al., 2005; Piera-Velazquez et al., 2020; Shi et al., 2020).

*IGF1* gene encodes insulin-like growth factor 1 that is similar to insulin in function and structure. IGF1 participates in the regulation of a variety of physiological processes, such as the regulation of cell cycle, mitosis, apoptosis, inflammation, and immunity (Guevara-Aguirre, 1996; Stewart and Rotwein, 1996; Heemskerk et al., 1999). Many studies have indicated that IGF1 protein was involved in the development of SSc (Hamaguchi et al., 2008; Winsz-Szczotka et al., 2016; Tabata et al., 2021). For instance, Hamaguchi et al. reported that patients with SSc had significantly increased levels of serum IGF1 compared with patients with systemic lupus erythematosus or healthy controls. In addition, the expression levels of serum IGF1 were even higher in patients with high skin thickness scores or with severe pulmonary fibrosis (Hamaguchi et al., 2008).

The EDN1, encoded by *EDN1* gene, is a secreted peptide that belongs to the endothelin/sarafotoxin family, and numbers of studies have illustrated the active role of EDN1 in fibrogenesis. EDN1 can lead to the conversion of fibroblasts to myofibroblasts and increase the deposition of excessive collagens, which are two key processes of tissue fibrosis (Shi-Wen et al., 2004, 2007; Soldano et al., 2009). Moreover, EDN1 can induce the apoptosis resistance in lung fibroblasts and can mediate the transforming growth factor-β (TGF-β)-induced fibrosis (Kulasekaran et al., 2009). In patients with SSc-ILD, the expression of EDN1 is increased in lung tissues, and the blockade of EDN1 could be an effective method to limit the progression of lung fibrosis (Abraham et al., 1997; Swigris and Brown, 2010).

*FOS* gene encodes c-FOS protein, which can dimerize with JUN family proteins to form transcription factor complex activator protein-1 (AP-1). The c-FOS proteins are included in many physiological processes, such as proliferation, differentiation, and transformation, and can act as regulators in tissue fibrosis (Angel and Karin, 1991; Sullivan et al., 2009). Importantly, in SSc, Avouac et al. found the upregulation of c-FOS in SSc animal models and the skin and dermal fibroblasts of SSc patients. Selective inhibition of AP-1 could decrease the production of collagens in SSc fibroblasts and prevent the development of experimental dermal fibrosis (Avouac et al., 2012).

To be mentioned, the lung fibrosis in SSc-ILD is caused by the interactions of multiple factors, such as immune cell infiltration, autoimmunity, inflammation, and vascular injury. Both the innate and adaptive immunity are involved in the pathogenesis of SSc-ILD, and immune cell infiltration is a hallmark of SSc-ILD (Furue et al., 2017; Brown and O’Reilly, 2019). In the current study, we evaluated the composition of infiltrating immune cells in lung tissues of SSc-ILD patients and identified differentially expressed immune cells between SSc-ILD patients and normal controls, such as M2 macrophages, DCs, and mast cells.

The polarization of M2 macrophages is closely related to the pathogenesis of tissue fibrosis in SSc (Chia and Lu, 2015; Manetti, 2015; Stifano and Christmann, 2016). M2 macrophages can produce profibrotic cytokine TGF-β, which plays a pivotal role in tissue fibrosis (Hu et al., 2018; Shapouri-Moghaddam et al., 2018). Christmann et al. (2014) compared the gene expression levels of lung tissues between SSc-ILD patients and controls, and the results revealed that M2 macrophage markers, such as *CD163* and *CCL18*, were upregulated in SSc patients, which were related to the progression of pulmonary fibrosis. Pechkovsky et al. (2010) illustrated the importance of M2 macrophage phenotype in the pathogenesis of lung fibrosis, and IL-4 and IL-10 could induce the shift of alveolar macrophages to M2. Our results in this study also found that the number of M2 macrophages was upregulated in lung tissues of SSc-ILD patients, which was consistent with those previous studies.

DCs are potent antigen-presenting cells (APCs) and play important roles in both innate and adaptive immune system (Audiger et al., 2017). Recently, the relationship between the pathogenesis of SSc and the expression of DCs, especially plasmacytoid DCs (pDCs), has been widely discussed (Lu, 2012; Affandi et al., 2018). The pDCs, as a special subset of DCs, could mediate the development of SSc by producing type I interferon (IFN-I) and other inflammatory mediators, such as chemokine C-X-C motif ligand 4 (CXCL4). To be mentioned, pDCs isolated from SSc patients perform the increased secretion of CXCL4, and the expression levels of CXCL4 in the plasma of SSc patients are correlated with the severity of fibrosis (van Bon et al., 2014; Ah Kioon et al., 2018). Moreover, Kafaja et al. (2018) found that the pDC levels were increased in the lung tissues but reduced in the peripheral blood of SSc patients compared with normal controls. In the bleomycin-induced fibrosis models, the depletion of pDCs could reduce the collagen deposition in lung and skin tissues and downregulate the expression of profibrotic genes, such as *TGF*β*1* (Kafaja et al., 2018). Overall, pDCs are involved in the pathogenesis of tissue fibrosis in SSc and may act as a potential therapeutic target for SSc patients.

Mast cells have also been reported in the pathogenesis of tissue fibrosis, and the skin of SSc patients has a relatively increased number of mast cells (Seibold et al., 1990). In addition, the mast cell numbers are also elevated in lung tissues of patients with fibrotic lung diseases, such as idiopathic pulmonary fibrosis (IPF) (Pesci et al., 1993; Veerappan et al., 2013). Mast cells can not only produce but also transfer the TGF-β, thus participating in the process of fibrosis (Hugle et al., 2011). Besides, other immune cells have also been identified to be involved in the complex immunopathogenesis of SSc, such as NK cells and neutrophils (Barnes et al., 2012; Almeida et al., 2015; Benyamine et al., 2018). However, in this study, the analysis of immune cell infiltration in lung tissues of SSc-ILD patients was based on the CIBERSORT algorithm, which only included 22 immune cell types. Considering the heterogeneity and complexity of the immune microenvironment, further studies are required to investigate the complete landscape of infiltrating immune cells in lung tissues of SSc-ILD. Nowadays, the rapid development of single-cell RNA-sequencing (scRNA-seq) technology provides us a novel method to explore the complex immune microenvironment. For instance, by conducting scRNA-seq analysis, Valenzi et al. (2019) identified multiple immune cell populations from 5 healthy control and 8 SSc-ILD lung tissue samples and demonstrated the fibroblast heterogeneity in SSc-ILD. Uncovering the heterogeneity of immune cell subpopulations in SSc-ILD and healthy lungs may provide new insights into the pathological mechanisms driving fibrosis in SSc-ILD.

In order to explore the potential regulatory mechanisms of genes on infiltration immune cells, we performed correlation analysis between core mRNAs and differential immune cells in lung tissues of SSc-ILD patients. The results showed that some fibrosis-related genes were significantly related to infiltrating immune cells (*P* < 0.001 and correlation coefficient > 0.5). For instance, the expression of *ACVRL1* gene was positively correlated with neutrophils and resting NK cells but negatively correlated with plasma cells. Interestingly, the correlation analysis presented in the current study also showed that the expression of *ACVRL1* gene was negatively correlated with the expression of *COL1A1* gene (*R* = −0.44) and *IGF* gene (*R* = −0.48), although these three genes were all fibrosis-related genes. *ACVRL1* gene encodes activin receptor-like kinase 1 (ALK1), which is one of the type I cell surface receptors for TGF-β (Munoz-Felix et al., 2013). Previous studies have proved that TGF-β is a profibrotic cytokine involved in numerous fibrotic disorders including SSc. In most cell types, TGF-β plays its profibrotic roles via TGF-β/ALK5/Smad2/Smad3 signaling pathway, which is the canonical TGF-β signaling pathway (Verrecchia and Mauviel, 2007; Lafyatis, 2014). Specifically, the fibrotic reaction is characterized by the increased synthesis of ECM proteins including type I collagen, the activation of fibroblasts, and the epithelial-mesenchymal transition (EMT). Smad 3 is indispensable for these processes, and *Smad3*-knockout mice are resistant to TGF-β- and bleomycin-mediated lung fibrosis (Willis and Borok, 2007; Santibanez et al., 2011). Besides, recent studies have also demonstrated that TGF-β could participate in the process of fibrosis via another ALK1/Smad1/Smad5 signaling pathway. However, the role of this pathway in fibrosis remains controversial (Munoz-Felix et al., 2013). On the one hand, ALK1/Smad1/Smad5 signaling pathway can regulate the profibrotic phenotype in SSc fibroblasts, leading to the increased production of ECM components, such as type I collagen and connective tissue growth factor (CTGF) (Pannu et al., 2007, 2008). On the other hand, ALK1 signaling can oppose the ALK5 pathway and inhibit the synthesis of ECM proteins in endothelial cells (Goumans et al., 2003; Finnson et al., 2008; Vorstenbosch et al., 2017). Therefore, the regulatory effect of ALK1/Smad1/Smad5 signaling on fibrosis might depend on the types of organs and cells, as well as other circumstances. This may partly explain the negative correlation between *ACVRL1* gene and some other fibrosis-related genes, but the detailed mechanisms need to be further studied. In addition, other core mRNAs, such as *COL1A1*, *FOS*, and *EDN1*, were positively or negatively correlated with the number of infiltrating immune cells including neutrophils, NK cells and mast cells. This correlation analysis gives us a novel insight into the gene regulatory mechanisms on immune system, and further studies are needed to investigate them in depth.

However, there are several limitations in this study. First, this study was conducted only based on the GEO database. Although we integrated four different microarray datasets of SSc-ILD, the sample size was still limited, partly because of the low morbidity of SSc-ILD. And not all the patients with SSc-ILD experienced lung biopsy, which could be another reason for the small sample size. Second, no lncRNA microarray dataset related to SSc-ILD was existed in the GEO database, so the potential target lncRNAs of DEmiRs were predicted using the online tool, which needs to be experimentally validated. Third, the analysis of immune cell infiltration was based on the CIBERSORT algorithm, which only included 22 immune cell types. Since the heterogeneity and complexity of the immune microenvironment were not taken into account, further studies are needed to investigate the complete landscape of infiltrating immune cells in lung tissues of SSc-ILD. Finally, considering that this is a pure bioinformatics analysis, the underlying regulatory mechanisms of the ceRNA network and gene-immune cells were not clearly elucidated, and further functional biological experiments with larger sample sizes are required.

To our best knowledge, this is the first comprehensive study to construct the potential ceRNA regulatory network and analyze the composition of infiltrating immune cells in lung tissues of SSc-ILD patients. Based on one miRNA and three mRNA microarray datasets, we identified DEmiRs and DEMs between SSc-ILD lung tissues and normal lung tissues, which were used for the construction of potential ceRNA regulatory network. Moreover, we also evaluated the differentially expressed infiltrating immune cells in lung tissues of SSc-ILD compared with normal controls and their correlation with core mRNAs, which provide new insights into gene-immune cells regulatory mechanisms in SSc-ILD. Our findings improve our understanding of the pathogenesis of SSc-ILD and may provide potential therapeutic targets for patients with SSc-ILD, which need to be further studied.

## Data Availability Statement

The datasets presented in this study can be found in online repositories. The names of the repository/repositories and accession number(s) can be found in the article/Supplementary Material.

## Author Contributions

YiL contributed to conception and design of the study. QW and YaL performed data analysis of the study. QW, YX, and SW contributed to the software analysis. QW prepared the original manuscript. QW, YaL, and YiL reviewed and edited the manuscript. All authors contributed to manuscript revision, read, and approved the submitted version.

## Conflict of Interest

The authors declare that the research was conducted in the absence of any commercial or financial relationships that could be construed as a potential conflict of interest.

## Footnotes

1 https://www.ncbi.nlm.nih.gov/geo

2 http://string-db.org

3 https://bioinfogp.cnb.csic.es/tools/venny/index.html

4 http://carolina.imis.athena-innovation.gr/diana_tools/web/index.php?r=lncbasev2/index

5 http://cibersort.stanford.edu/
